# Cardiac Resynchronization Therapy in Non-Ischemic Cardiomyopathy: Role of Multimodality Imaging

**DOI:** 10.3390/diagnostics11040625

**Published:** 2021-03-30

**Authors:** Cristian Stătescu, Carina Ureche, Ștefana Enachi, Rodica Radu, Radu A. Sascău

**Affiliations:** 1 Cardiology Department, Cardiovascular Diseases Institute “Prof. Dr. George I.M. Georgescu”, Carol I Boulevard No. 50, 700503 Iași, Romania; cstatescu@gmail.com (C.S.); stefana.enachi@yahoo.com (Ș.E.); rodiradu@hotmail.com (R.R.); radu.sascau@gmail.com (R.A.S.); 2 Internal Medicine Department, “Grigore T. Popa” University of Medicine and Pharmacy, 700115 Iași, Romania

**Keywords:** cardiac resynchronization therapy, non-ischemic cardiomyopathy, echocardiography, cardiac magnetic resonance, myocardial dyssynchrony

## Abstract

Non-ischemic cardiomyopathy encompasses a heterogeneous group of diseases, with a generally unfavorable long-term prognosis. Cardiac resynchronization therapy (CRT) is a useful therapeutic option for patients with symptomatic heart failure, currently recommended by all available guidelines, with outstanding benefits, especially in non-ischemic dilated cardiomyopathy. Still, in spite of clear indications based on identifying a dyssynchronous pattern on the electrocardiogram (ECG,) a great proportion of patients are non-responders. The idea that multimodality cardiac imaging can play a role in refining the selection criteria and the implant technique and help with subsequent system optimization is promising. In this regard, predictors of CRT response, such as apical rocking and septal flash have been identified. Promising new data come from studies using cardiac magnetic resonance and nuclear imaging for showcasing myocardial dyssynchrony. Still, to date, no single imaging predictor has been included in the guidelines, probably due to lack of validation in large, multicenter cohorts. This review provides an up-to-date synthesis of the latest evidence of CRT use in non-ischemic cardiomyopathy and highlights the potential additional value of multimodality imaging for improving CRT response in this population. By incorporating all these findings into our clinical practice, we can aim toward obtaining a higher proportion of responders and improve the success rate of CRT.

## 1. Introduction

Non-ischemic cardiomyopathy encompasses a heterogeneous group of diseases, mostly with genetic determinism, with a generally unfavorable long-term prognosis due to the evolution to pump failure and sudden arrhythmic death, secondary to limited therapeutic options.

Cardiac resynchronization therapy (CRT) is a useful therapeutic method for patients with heart failure, improving cardiovascular prognosis. The first set of evidence for the clinical benefits of CRT comes from the Multicenter InSync Randomized Clinical Evaluation (MIRACLE) trial, which showed a significant improvement in symptoms of patients with heart failure and left ventricle ejection fraction (LVEF) < 35%, with a QRS duration over 130 ms [1]. Subsequently, the Comparison of Medical Therapy, Pacing, and Defibrillation in Heart Failure (COMPANION) and Cardiac REsynchronization in Heart Failure (CARE–HF) trials demonstrated benefits on morbidity and all-cause mortality, both in ischemic and non-ischemic patients [2,3].

These studies established the scientific bases for the 2016 European Society of Cardiology Heart Failure Guidelines that recommend CRT for patients with an LVEF below 35%, symptomatic despite optimal medical treatment with beta-blockers, angiotensin-converting enzyme inhibitors or angiotensin receptor blockers, aldosterone antagonists, and diuretics, in sinus rhythm, with a QRS duration of over 120 ms and left bundle branch block (LBBB) or non-LBBB morphology [4].

When discussing CRT response, it is important to distinguish between cardiac reverse remodeling, which is frequently assessed by imaging, and the net clinical benefit, with impact on hard endpoints such as all-cause death or hospitalization for heart failure. In this regard, first reported in 2005 [5], the greater benefit of CRT on cardiac remodeling and clinical endpoints in non-ischemic patients was observed in sub-analyses of all major clinical trials: Multicenter InSync Randomized Clinical Evaluation (MIRACLE) [6], Cardiac Resynchronization in Heart Failure (CARE–HF) [7], Resynchronization Reverses Remodeling in Systolic Left Ventricular Dysfunction (REVERSE) [8], and Multicenter Automatic Defibrillator Implantation with Cardiac Resynchronization Therapy (MADIT–CRT) [9]. On the other hand, patients with ischemic cardiomyopathy exhibit less cardiac remodeling, present more adverse events, and have a worse prognosis after CRT. Still, those who do exhibit cardiac remodeling have a 0.9% relative risk reduction for each 1% reduction in left ventricle (LV) end-systolic volume and an absolute risk reduction of 0.4%, similar to those with non-ischemic cardiomyopathy [10].

Together with echocardiography, over the last few years, new imaging modalities such as cardiac magnetic resonance (CMR) and nuclear imaging have proved their value in assessing mechanical dyssynchrony. Moreover, we anticipate that many new parameters will continue to emerge as tools to predict, guide, and assess CRT response. There are other papers that have addressed the contribution of imaging to this field, but this review provides an up-to-date synthesis of the latest evidence, with a focus on non-ischemic cardiomyopathy. Additionally, we discuss the hypothesis-generating studies that translated into trials examining hard clinical endpoints. By doing so, this paper highlights the potential additional value of multimodality imaging for improving CRT response and its clinical relevance in everyday practice. Still, future randomized control trials are needed in order to conclude if this approach can ultimately reduce costs and lead to a net clinical benefit.

## 2. Cardiac Resynchronization in Cardiomyopathies

Non-ischemic dilated cardiomyopathy: Among cardiomyopathies, the greatest benefit of CRT is proven in non-ischemic dilated cardiomyopathy (DCM); see Table 1.

The MIRACLE trial included 113 patients with non-ischemic cardiomyopathy and 115 patients with ischemic cardiomyopathy. At baseline, the non-ischemic group had bigger LV volumes and lower LVEF when compared to the ischemic group (325.2 ± 128.2 mL vs. 284.1 ± 84.5 mL and 23.2 ± 7.2% vs. 24.8 ± 6.3%, respectively). Interestingly, at 12 months after CRT implantation, the non-ischemic group exhibited a greater reduction in LV volumes and improvement in LVEF than those in the ischemic group (275 ± 138 mL vs. 278 ± 87 mL and 33.1 ± 12.6% vs. 29.5 ± 9.9%, respectively). Additionally, patients in the non-ischemic group had a higher quality of life at 12 months after CRT implantation [6].

The CARE–HF trial included 339 patients with ischemic heart disease and 473 patients with non-ischemic cardiomyopathy. The primary endpoint (all-cause mortality or unplanned hospitalization for a major cardiovascular event) was significantly reduced in the non-ischemic group, compared to the ischemic population (HR 0.46 vs. 0.71). Still, there was an important benefit for both populations in terms of the secondary outcome (all-cause mortality)—HR 0.56 vs. 0.54 [7].

In the REVERSE trial, 610 patients were randomized to CRT (*n* = 419; 236 ischemic and 183 non-ischemic) or medical therapy (*n* = 191; 97 ischemic and 94 non-ischemic). At 12 months of follow up, there was a more important improvement in all the echocardiographic parameters, from a threefold increase in LVEF (+7.61% vs. +2.24%) to a reduction in mitral regurgitation (MR) and improvement in diastolic dysfunction [8].

In MADIT–CRT, 1046 ischemic and 774 non-ischemic patients were investigated comparatively over a period of 2.4 years, showing a 34% reduction in the risk of heart failure and mortality (*p* = 0.001) among ischemic patients and a 44% reduction among non-ischemic patients (*p* = 0.002). In addition, it was observed that among non-ischemic patients there was a greater reduction in both end-systolic (37 ± 16%) and end-diastolic volume (24 ± 12%, *p* < 0.001), with better response to CRT in women, diabetic patients, and those with an LBBB morphology [9]. The benefits of resynchronization translate into a survival benefit—55% at 4 years for ischemic vs. 77% for non-ischemic patients [11].

Hypertrophic cardiomyopathy (HCM): A minority of patients with HCM (5%) end up developing a dilated form (end-stage HCM), characterized by increased volumes, spherical remodeling, and severe functional impairment of LV defined by an LVEF < 50% [12].

The first data on the benefit of biventricular pacing in HCM appeared in 2008 when Rogers et al. have investigated a group of 20 patients with CMH and LBBB and showed that biventricular pacing reduces the dimensions of the left atrium and left ventricle and can relieve the symptoms of heart failure [13]. Moreover, there are data suggesting that CRT can bring short-term benefits and gain time to heart transplantation, improving patients’ quality of life without additional risks [14,15]. Still, a study that included 16 patients with dilated phase HCM, 231 patients with idiopathic DCM, and 65 patients with ischemic DCM showed that patients with dilated HCM respond poorly to CRT and have an unfavorable long-term prognosis [16]. However, all these data must be confirmed by further studies.

Another utility of CRT in patients with HCM may result from the diminution of the obstruction in left ventricular outflow tract (LVOT). In this regard, a pilot study published in 2011 that included 11 patients with hypertrophic obstructive cardiomyopathy (HOCM) with indication of implantable cardiac defibrillator (ICD) opted for the implantation of a CRT-D system. After six months of pacing, a reduction in NYHA class, peak and mean gradient was observed (33 vs. 84 mmHg and 13 vs. 38 mmHg, respectively). In six patients, there was a reduction of more than 50% in the gradient, which fell below 40 mmHg [17].

The Triple Chamber Pacing in Hypertrophic Obstructive Cardiomyopathy Patients (TRICHAMPION) trial is a prospective, single-blinded trial that included 25 patients and aimed to investigate the benefit of CRT-P in symptomatic patients with HOCM and severe LVOT obstruction, who were not candidates for ablative therapy. The primary endpoint was the relief of symptoms. The enrollment was closed in 2020, but the results have not yet been published [18].

Left ventricular noncompaction cardiomyopathy (LVNC): LV noncompaction is frequently associated with the presence of heart failure and systolic dysfunction. A systematic review that identified 14 studies and 70 patients reported by 2018 showed, however, that CRT brings clinical benefits to these patients, and that those who respond to therapy benefit from improved systolic function, leading to improved contractility of noncompacted segments [19]. This observation was later confirmed by single photon emission computed tomography (SPECT) imaging [20]. However, it seems that in this subpopulation, it is very important to highlight the intraventricular since it correlates better with the CRT response than the duration of the QRS complex [21].

Cardiac sarcoidosis: Data on CRT for patients with cardiac sarcoidosis are scarce. A study that included 19 patients with cardiac sarcoidosis who underwent CRT showed that there is a benefit for this category of patients, with the improvement of the LVEF from 28.8% to 35.9% (*p* < 0.05), but without reduction in the end-diastolic and end-systolic volumes, improvement of right ventricular function or reduction of mitral regurgitation [22]. However, it appears that compared to patients with idiopathic DCM, patients with cardiac sarcoidosis respond less to CRT, the incidence of major adverse cardiovascular and cerebrovascular events (MACCE) is higher [23], and they have a higher incidence of ventricular arrhythmias [24].

## 3. Multimodality Imaging of Myocardial Dyssynchrony

The principle behind cardiac resynchronization is cardiac dyssynchrony, and the majority of the benefits of this therapy lie in its improvement. The Results of the predictors of response to CRT (PROSPECT) trial analyzed multiple echocardiographic parameters indicative of asynchronism by M mode and tissue Doppler imaging, which did not demonstrate benefits in predicting CRT response. The investigators considered that the negative results resided in the intra- and interobserver variability [25]. Subsequently, The Speckle Tracking and Resynchronization (STAR) study and the TARGET (Targeted Left Ventricular Lead Placement to Guide Cardiac Resynchronization Therapy) trial have shown that speckle tracking echocardiography may be a predictor of CRT response [26,27].

Atrioventricular dyssynchrony: this is present and significant when atrial systole occurs much earlier than ventricular systole. This causes early and incomplete closure of the mitral valve, with the onset of diastolic mitral regurgitation and shortening of the filling time. Thus, atrial systole no longer contributes to diastolic filling. The ratio between filling time and the RR distance of <40% is significant. The presence of diastolic mitral regurgitation is an indicator of atrioventricular asynchronism and correlates with an unfavorable response to CRT [28].

Interventricular dyssynchrony: this can be quantified by pulsed-wave Doppler and tissue Doppler imaging by measuring the time difference between the closure of the aortic and pulmonary valve (with a cut-off value of 40 and 56 ms, respectively).

Intraventricular dyssynchrony: this represents a fundamental concept in CRT, and there are several parameters that suggest its presence.

“True” LBBB: In patients with an LBBB on the electrocardiogram, it was observed that the myocardial deformation map differs depending on the etiology of the conduction disorder. Thus, in patients with ischemic LBBB, an initial septal contraction is observed with subsequent propagation to the lateral wall, while in patients with non-ischemic LBBB, there is an interruption of propagation in the anterior wall that contracts later, thus determining a U-shape activation (“horseshoe”). This latter type of activation is associated with a better response to CRT, and the identification of patients with “true” LBBB is important because they benefit the most from CRT.

Over time, it has been observed that a small proportion of patients with idiopathic DCM respond very well after CRT—the so-called hyper responders—who achieve complete normalization of LVEF after resynchronization. This observation suggests that the presence of LBBB may be the causal factor of DCM for these patients [29]. However, in the presence of a patient who is diagnosed with DCM and has LBBB on the ECG, it is difficult to differentiate whether LBBB is the cause or consequence of DCM. In this regard, a study of 63 non-ischemic patients with DCM and LBBB showed that CMR can differentiate patients in whom LBBB preceded DCM, who exhibit a preserved systolic function in the lateral wall, as opposed to those in whom LBBB occurred later. This study further confirmed that resynchronization in these patients brings survival benefits, especially since the development of LBBB is an independent predictor of mortality for patients with idiopathic DCM [30,31].

**Figure 1:**
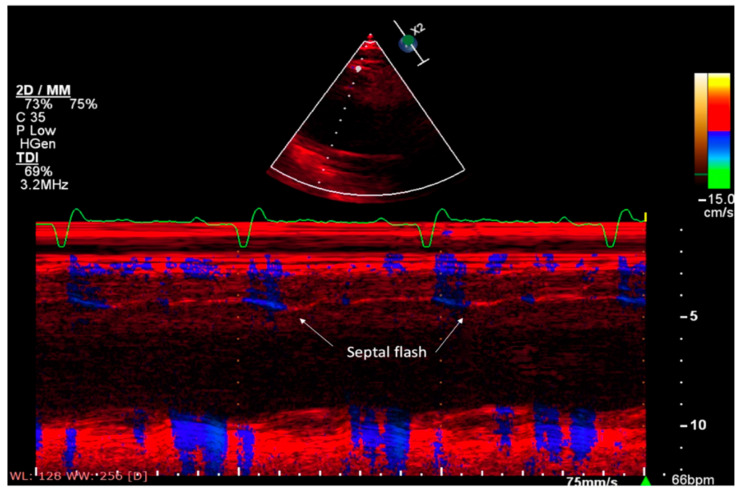
Septal flash in a patient with DCM, candidate for CRT (authors’ personal collection).

Septal flash: In the context of LBBB, the first site of activation of the LV endocardium is called “the breakthrough site” and is located in the mid-apical septum. Therefore, the first notch on the surface ECG reflects the activation of the septal endocardium of the LV, and the second, the activation of the posterolateral wall. Since the free wall is not yet activated in the protosystole, the intraventricular pressure does not increase and the septum may contract at this breakthrough point. Later, when the pressure increases, the septum is pushed to the right. This early septal contraction is called “septal flash” and is associated with passive relaxation of the inferolateral wall, followed by contraction of this wall during passive relaxation at the septal level (Figure 1). This pattern causes inefficient mechanical contraction [32].

Apical rocking: Apical rocking, a movement identified in the apical four-chamber view, is characterized by a short septal movement of the apex due to early contraction of the septum in systole, followed by a longer lateral movement during ejection due to late contraction of the lateral wall, secondary to the presence of LBBB. This clockwise movement is typical of idiopathic DCM and indicates the presence of intraventricular asynchronism. The magnitude of the movement is directly proportional to the LV end-diastolic volume and is not influenced by the duration of the QRS complex. For non-ischemic patients, the presence of apical rocking predicts a benefit from CRT [33]. Recently, the Markers and Response to CRT (MARC) trial, which included 240 patients with heart failure (46% of ischemic origin), showed that apical rocking and the presence of interventricular delay are the only echocardiographic parameters able to predict a positive reverse remodeling, expressed as LV end-systolic volume change [34].

Furthermore, 3D speckle-tracking echocardiography contributes to a better understanding of the pathophysiology of intraventricular asynchronism. A study that included 65 patients, of whom 20 with DCM and LVEF < 35% and QRS over 120 ms, 20 with DCM and LVEF < 35% and QRS below 120 ms, and 25 controls, used as indicators of intraventricular asynchronism the standard derivation of time to peak of the radial by 3D speckle tracking and time to peak rotation by 2D and 3D speckle tracking. It was observed that compared to control subjects, all patients with DCM exhibited an alteration of rotational indexes, the most affected being those with wide QRS, while LV torsion in patients with DCM and wide QRS was lower than in those with DCM and narrow QRS complex. Moreover, LV torsion after resynchronization was markedly improved in those with DCM and wide QRS, with a reduction in intraventricular asynchronism [35].

CMR allows the evaluation of intraventricular dyssynchrony by three methods: myocardial tagging, displacement encoding with stimulated echoes (DENSE), and phase-contrast tissue velocity mapping [36].

In 2013, Cochet et al. evaluated cardiac dyssynchrony on CMR in order to predict ventricular remodeling after CRT implantation. Asynchronism based on radial displacement and delayed enhancement data were used, while reverse remodeling was defined as a reduction in LV end-systolic volume by >15% at six months after device implantation. The study showed that the presence of intraventricular asynchronism independently predicted reverse remodeling, with a sensitivity of 67% and a specificity of 75%. Moreover, the identification of scar tissue by late gadolinium enhancement (LGE) was an independent predictor of the response, which was weak when the LV lead was placed at the level of the ventricular scar. Additionally, the presence of asynchronism was a more important predictor of CRT response than the duration of the QRS complex [37].

Myocardial perfusion SPECT also predicts the response to CRT among patients with non-ischemic DCM with a sensitivity of 86% and a specificity of 80% [38]. In addition, SPECT may be useful in identifying the most suitable segments for pacing, proving that LV pacing in the segments with the latest contraction and the latest relaxation has the potential to improve the CRT response rate [39].

A complete list of multi modality imaging parameters validated for CRT guidance can be found in Table 2.

## 4. Multimodality Imaging of the Myocardial Substrate

A study published in 2015 by Lumens et al. started from the hypothesis that the difference in response between the ischemic and non-ischemic populations is based on the type of substrate. The study included 191 patients and showed that in the case of a true LBBB, the electromechanical substrate is a delay in conduction and thus the contraction of the posterolateral segment of up to 350 ms, which explains why these patients respond to CRT. In patients with a hypo contractility pattern in the lateral wall secondary to ischemic heart disease, the delay is not caused by a slowing in the propagation of electrical activation, but by the hypo contractile condition itself. This subgroup of patients benefits less from CRT and most often does not have a typical LBBB morphology, but rather nonspecific intraventricular conduction disorder [40]. In the extreme case of patients with a transmural fibrous scar at the posterolateral wall, CRT does not lead to any benefit because the scar cannot be recruited in the ventricular contraction, even despite the presence of a QRS complex large enough to meet the guidelines criteria.

Therefore, it derives the need for a detailed analysis of the myocardial substrate before CRT implantation. This can be performed by echocardiography and CMR. Unlike ischemic cardiomyopathy, in which contractile asynchronism can derive from the presence of transmural or subendocardial scars that lead to hypokinetic or akinetic segments, non-ischemic patients often have a pattern of mid-myocardial or subepicardial scarring.

By dobutamine stress echocardiography, one can assess the left ventricular contractile reserve (LVCR), a parameter that is independently associated with reverse remodeling after CRT. A meta-analysis that included eleven studies and 861 patients showed that LVCR can predict a favorable CRT response (OR 2.06 (95%, CI 1.70–2.43), Z score: 11.055, *p* < 0.001) [41].

Myocardial deformation imaging allows more accurate localization of scarred areas, which most often correlate with the localization obtained on CMR. Myocardial strain, longitudinal (GLS), radial (GRS), and circumferential (GCS), is a measure of myocardial deformation that is reduced in the presence of diseased myocardium. Specific cut-offs have been described for identifying the presence of a transmural scar as follows: <−10% for GLS, <−16.5% for GRS, and >−11.1% for GCS. In addition to predicting the myocardial substrate, Delgado-Montero et al. showed that values of GLS < −9% and GCS > −9% are associated with a higher risk of hospitalization, death, the necessity for LV assist device or heart transplantation, independent of CRT implantation [42]. Moreover, GLS values correlate with the presence of transmural scars on CMR, and a value of GLS of −4.5% differentiates between segments with viable myocardium and those with scars on CMR with a sensitivity of 81.2% and a specificity of 81.6% [43].

The presence of ventricular mid-myocardial fibrosis detected by CMR is an independent predictor of mortality for patients with DCM, both by pump deficiency and sudden cardiac death [44]. Compared to echocardiography, CMR offers the benefit of a very good temporal and spatial resolution that allows the precise identification of post MI ischemic scars by the presence of LGE. CMR may also be useful in selecting those patients who may benefit from a CRT-D system because there is insufficient data to support CRT-D implantation for all non-ischemic patients with a CRT indication. In this regard, there are data suggesting that some of the features of myocardial scars on CMR such as extension or heterogeneity correlate with the need for ICD therapies in these patients [45]. A study that included 48 patients with an indication for resynchronization showed that a scar burden percentage of over 13.7% of LV mass is the only independent predictor of the absence of post-CRT myocardial remodeling. In the same study, diffuse interstitial fibrosis was not a predictor of CRT response [46].

A study published by Bleeker et al. that studied 40 patients with LVEF < 35%, QRS > 120 ms, and LBBB morphology, and showed that those with posterolateral scar identified on CMR are mostly non-responders, regardless of the presence or absence of contraction dyssynchronism, while over 80% of the patients without scar respond to CRT [47]. Levya et al. recently demonstrated on a population of 559 resynchronized patients, 34% non-ischemic, with a mean QRS duration of 154 ± 28 ms, and followed for nine years, that the 43 patients who had scar present on CMR had had a clearly unfavorable prognosis, compared to those without (highest risk of CV mortality—HR 6.34, CV mortality or hospitalization for heart failure—HR 5.57, death from any cause or hospitalization for major adverse cardiovascular events (MACE)—HR 4.74, all with *p* < 0.0001) [48].

Another important study that included 75 patients who underwent CMR before the CRT implantation was published in 2014. Strain values based on the circumferential uniformity ratio (CURE) on cine DENSE were used to obtain scar data. In addition, mechanical stretch at the site of the LV probe and electrical activation delay was measured. After a follow-up period of 2.6 years, all parameters were predictors of CRT response, and a CURE index > 0.7 was associated with a 12-fold higher risk of death [49].

## 5. Classical Echocardiographic Parameters Useful Pre-CRT

Apart from the aforementioned cases, prior to the implantation of the resynchronization system, echocardiography can also be used to measure EF and ventricular volumes to determine the existence of wall motion abnormalities and quantify the severity of mitral regurgitation.

LVEF before resynchronization may predict CRT response in patients with non-ischemic DCM. In a 2019 study, in which patients were followed by 2D and 3D echocardiography before and after implantation, it was observed that at an LVEF < 22.15%, no benefit was recorded after CRT, and in addition, the presence of mechanical asynchronism in these patients did not provide additional value to improve the patient selection process [50]. Discordantly, in 2021, the Cleveland Clinic and the Johns Hopkins Hospital report in The Journal of the American College of Cardiology that there could be a benefit even in the case of an LVEF below 15% for about one-third of patients, inversely proportional to the degree of dilation and the duration of the QRS complex [51]. Certainly, the progress made in the resynchronization technique and the medical treatment of heart failure in the last years has a very important role.

The degree of left ventricular dilation before resynchronization is an important predictor of post-implant remodeling, LVEF changes, and survival [52]. An LVEDV > 255 mL (OR = 2236; 95% CI, 1016–4923) predicts the lack response to CRT [53].

A study that included 100 non-ischemic patients with an indication for CRT showed that the presence of diastolic dysfunction before resynchronization can influence the response to CRT. Thus, patients with a delayed relaxation type 1 profile benefit the most, for those with a pseudonormal profile the response is more delayed, and patients with a restrictive type 3 profile do not benefit from the implant [54]. An E/A > 1 (OR = 0.211; 95% CI, 0.079–0.566) and e’/a’ > 1 (OR = 0.054; 95% CI, 0.017–0.172) predict a high probability for a positive CRT response [53]. Additionally, it appears that CRT in patients with idiopathic DCM prevents the onset of atrial fibrillation, reduces left atrial volume, and improves diastolic dysfunction [55,56].

In DCM, mitral regurgitation (MR) is favored by dilation of the heart cavities and by modification of the architecture of the mitral valve apparatus, especially the posterior displacement of the anterolateral papillary muscle. CRT reduces the degree of MR, which is associated with a longer period without the need for transplantation [57]. The main determinants of early improvement in MR are a reduction in the height of the coaptation area and an improvement in intraventricular asynchronism, while the determinants of MR reduction at six months are represented by the restoration of the initial position of the anterolateral papillary muscle and the increase in the sphericity index [58].

Data on the effects of CRT on right ventricular dysfunction are contradictory. However, it appears that patients with right ventricular dysfunction and right intraventricular contraction asynchronism respond poorly to CRT, but this should not be considered an exclusion criterion [59,60].

## 6. Role of Multimodality Imaging for Guiding Lead Positioning

In theory, the identification of the areas with the latest activation (usually the lateral or inferolateral wall) by imaging could guide the positioning of the lead in order to succeed in recruiting the viable myocardium for the ventricular contraction to be as efficient as possible.

Initially, the data seemed promising as the Targeted Left Ventricular Lead Placement to Guide Cardiac Resynchronization Therapy (TARGET) trial, which included 220 patients, showed that guiding lead position by speckle-tracking imaging leads to an improvement in both echocardiographic (reduction of LV end-systolic volume) and clinical response [27]. A year later, the Speckle Tracking Assisted Resynchronization Therapy for Electrode Region (STARTER) trial used radial strain for lead positioning, demonstrating a clinical benefit for echo-guidance, with an improvement in event-free survival (HR 0.40; 95% CI, 0.22–0.71; *p* = 0.002) [61].

However, the most recent data come from the ImagingCRT trial, a clinical trial that randomized 182 patients (49% ischemic, 76% with a QRS > 150 ms) to undergo CRT with the lead positioned in the area with the latest activation, as identified by echocardiography or conventionally; the trial showed no benefit for those patients in whom the LV lead was positioned after ultrasound guidance [62]. Given the conflicting results, further studies are needed to draw a definitive conclusion in this regard.

Positioning the lead by CMR guidance could be promising. Still, a study that included 99 patients with an indication for CRT and followed for 47 months, showed that CMR guidance does not bring any benefit on the risk of cardiovascular death or hospitalization for heart failure compared to classical guidance. However, in the subgroup of patients with LBBB on ECG and class NYHA > II, the benefit was in favor of CMR guidance (HR = 0.09; 95% CI: 0.01–0.75) [63].

The SPECT Guided LV Lead Placement for Incremental Benefits to CRT Efficacy (GUIDE CRT) trial enrolled 194 consecutive patients from 19 centers in China who underwent a myocardial SPECT within seven days prior to resynchronization. In the SPECT-guided group, a better placement of the LV lead was observed (85.5% vs. 62.4%, *p* < 0.002), a more significant reduction of LVESV (48.2 ± 61.6 mL vs. 28.9 ± 54.6 mL, *p* < 0.030), of LVEDV (49.4 ± 67.9 mL vs. 17.7 ± 103.9 mL; *p* < 0.036), and an increase in the response rate (57.1% vs. 35%, *p* < 0.025) [64].

## 7. Multimodality Imaging for CRT Optimization

The benefits of cardiac resynchronization derive mainly from changes in the myocardial activation sequence. In the CArdiac REsynchronization in Heart Failure (CARE–HF) trial, resynchronization parameters after implantation were optimized using echocardiography, and currently, echocardiography is the most widely used method for optimization [3]. However, in daily practice, it is estimated that only 40% of resynchronized patients benefit from post-implant optimization, mostly due to the lack of trained staff [65].

For maximum optimization, the atrioventricular delay (AV delay) is set to obtain the maximum separation between the E and A waves on the transmitral pulsed-wave Doppler [66]. After proper optimization of the AV interval by obtaining the maximum separation of the E and A waves, the increase of the subaortic velocity time integral (VTI) and implicitly of the cardiac output is observed by recruiting the ejection volume obtained from the decrease of the mitral regurgitation.

System optimization can also be performed by setting the interventricular (VV) delay, an indicator of interventricular asynchronism. This can also be conducted by following the LVOT VTI by pulsed-wave Doppler and subsequent selection of the largest VTI. Both this method and tissue Doppler imaging can be used for optimization. For most patients, the most appropriate VV interval is LV pre-activation [67].

Still, there is an increasing tendency to look for alternative methods of post-implant optimization of the CRT system.

In a recent trial, 63 resynchronized patients were randomized to optimize the VV interval based on QRS complex duration (*n* = 31) or based on systolic asynchrony index derived from 3D echocardiography. At 12 months of follow up, no significant differences were observed on ventricular remodeling or clinical evolution of patients between the two groups [68].

Recently, promising data have emerged using the AdaptivCRT algorithm (Medtronic) [69] and methods based on implantable hemodynamic sensors (SonR) [70]. The main limitation of these methods is that they are intended for a single manufacturer.

In this regard, the Clinical Trial of the SonRtip Lead and Automatic AV-VV Optimization Algorithm in the PARADYM RF SonR CRT-D (RESPOND–CRT), which randomized 998 patients for optimization using echocardiography or a SonR contractility sensor located in the right atrium, allows the optimization of both AV and VV intervals. Overall, the response rate was 75% in the SonR arm and 70.4% in the echocardiography-guided arm (MD 4.6%, CI −1.4 to 10.6%, *p* < 0.001 for non-inferiority −10% margin). Moreover, the use of the SonR sensor was associated with a 35% reduction in hospitalizations for HF (HR 0.65, 95% CI 0.46–0.92; log rank *p* = 0.01) [70].

The British Randomized Controlled Trial of AV and VV Optimization (BRAVO; NCT01258829) randomized 401 resynchronized patients for optimization by echocardiography or hemodynamics multiple-replicated, beat-by-beat noninvasive blood pressure at baseline, aiming as primary endpoint peak exercise oxygen uptake at six months. The results showed that hemodynamic optimization is non-inferior to echocardiography, and through its potential for automation and reproducibility, it can serve as a useful tool for optimizing the CRT system [71].

Another recent study started from the hypothesis that an electrophysiological implant with a left ventricular lead placed at the level of the latest activated segment, followed by an optimization of the VV interval after the implant, is non-inferior to an implant performed based on CT venous angiography, myocardial scintigraphy with 82Rubidium infusion, and speckle-tracking echocardiography. Thus, the 122 patients were randomized in a double-blind manner, noting that the electrophysiological guidance is non-inferior to six months. However, larger studies are needed to evaluate the feasibility of a purely electrical-guided CRT implantation [72].

## 8. Conclusions

In conclusion, CRT has a proven benefit in non-ischemic cardiomyopathy, especially DCM. Echocardiography, CMR, and SPECT imaging can help with refining the patient selection criteria, the implant technique, and subsequent optimization of the system. The most important predictors of CRT response are the absence of mechanical asynchronism at the baseline, the presence of the scarring myocardium, and the suboptimal positioning of the left ventricular lead. In the years to come, we anticipate many new imaging parameters will continue to emerge in this field, and by incorporating some of them in our daily practice, we can aim toward obtaining a higher proportion of responders and improving the success rate of CRT. Future randomized control trials are needed in order to conclude if an imaging-based approach can ultimately reduce costs and contribute to a net clinical benefit.

## Figures and Tables

**Table 1:** Summary of studies investigating cardiac resynchronization therapy (CRT) in non-ischemic dilated cardiomyopathy (DCM).

Study	Type of Study	Comparison	Inclusion Criteria	No. of Patients	No. of Patients with Non-Ischemic Cardiomyopathy	Primary End-Points	Results Non-Ischemic vs. Ischemic	Conclusions
MIRACLE 2002 [6]	RCT	CRT in ischemic vs. non-ischemic	NYHA class III/IV LVEF ≤ 35% QRS ≥ 130 ms LVEDD ≥ 55 mm	228	113	NYHA class Echo parameters QoL score 6MWT	NYHA class 2.2 ± 0.8 vs. 2.2 ± 0.8 LVEDV, mL, 275 ± 138 vs. 278 ± 87 LVEF, % 33.1 ± 12.6 vs. 29.5 ± 9.9 LV mass, g 301 ± 103 vs. 299 ± 79 QoL score 39 ± 24 vs. 38 ± 23 6MWT, m 342 ± 120 vs. 318 ± 149	Greater benefit on echocardiographic and clinical parameters in non-ischemic cardiomyopathy
CARE-HF 2005 [7]	RCT	CRT vs. OMT	NYHA class III/IV LVEF ≤ 35% Cardiac dyssynchronyLVEDD ≥ 30 mm/m^2^	814	473	All-cause mortality or unplanned hospitalization for a major CV event	HR 0.46 (0.35–0.63) vs. 0.71 (0.54–0.94)	Greater benefit on primary end-point in non-ischemic cardiomyopathy
REVERSE 2008 [8]	RCT	CRT + OMT vs. OMT	NYHA class I/II LVEF ≤ 40% QRS ≥ 120 ms LVEDD ≥ 55 mm	610	183 CRT ON 94 CRT OFF	Echo parameters	LVEDVi, mL/m^2^ −30.5 vs. −10.7 LVEF, % +7.61 vs. +2.24 LV mass, g −24.1 vs. −11.5	Greater benefit on echocardiographic parameters in non-ischemic cardiomyopathy
MADIT–CRT 2009 [9]	RCT	CRT + OMT vs. ICD + OMT	NYHA class I/II LVEF ≤ 30% QRS > 130 ms	1820	774	Risk of HF or death First HF event Echo parameters	HF or death: 0.56 (0.39–0.80) vs. 0.66 (0.52–0.85) HF event: 0.50 (0.35–0.75) vs. 0.58 (0.45–0.77) LVEDV −24 ± 12% vs. −18 ± 10% LVEF 12 ± 5% vs. 10 ± 5%	Greater benefit on primary end-points in non-ischemic cardiomyopathy

CRT—cardiac resynchronization therapy; DCM—dilated cardiomyopathy; OMT—optimal medical therapy; ICD—implantable cardiac defibrillator; LVEF—left ventricular ejection fraction; LVEDV—left ventricular end-diastolic volume; LVEDD—left ventricular end-diastolic diameter; QoL score—quality-of-life score; 6MWT—six-minutes-walk test.

**Table 2:** Multimodality imaging for improving CRT response—validated parameters.

Application	Echocardiography	CMR	SPECT
Myocardial dyssynchrony	Filling time/RR distance < 40% Difference between projection time at the aortic and pulmonary level > 40 ms in PWD and 56 ms in TDI Septal flash—septal to posterior wall motion delay in M mode > 130 ms Apical rocking Septal to lateral delay in TDI > 60 ms Max delay in 4 basal LV segments in TDI > 65 ms SD of 6 basal LV segments in TDI > 36.5 ms Max delay in 12 basal and mid-LV segments in TDI > 100 ms SD of 12 basal and mid-LV segments (dyssynchrony index) in TDI > 32.6 ms SD of time-to-peak longitudinal strain in 12 basal and mid-LV segments in TDI color > 60 ms Anteroseptal to posterior time to peak strain difference (radial strain) in 2D speckle tracking > 130 ms SD of time to minimum systolic volume of 16 LV segments (systolic dyssynchrony index) > 5.6%	CMR myocardial tagging (similar to speckle tracking in echocardiography) Phase Contrast Tissue Velocity Mapping (similar to TDI in echocardiography) DENSE—measures strain directly CURE—index of circumferential strain	Timing of regional wall thickening during a cardiac cycle provided by phase analysis—PHD and PSD
Myocardial substrate	Indicators of transmural scar: GLS < −10%, GRS < −16.5% and GCS > −11.1% LV contractile reserve by dobutamine stress echo	Scar at LV pacing site by late gadolinium enhancement technique	- Scar at LV pacing site by T1 SPECT-MPI
Lead positioning	Pacing at the latest activation site identified by speckle-tracking echocardiography	Pacing at the latest activation site identified by CMR	Pacing at the latest activation site identified by SPECT
CRT optimization	AV delay—maximum separation of E and A waves by PWD VV delay—PWD at the level of the LVOT; systolic asynchrony index on 3D echocardiography		

CRT—cardiac resynchronization therapy; CMR—cardiac magnetic resonance; SPECT—single-photon emission computed tomography; TDI—tissue Doppler imaging; PWD—pulsed-wave Doppler; DENSE—displacement encoding with stimulated echoes; CURE—circumferential uniformity ratio; PHD—phase histogram bandwidth; PSD—phase distribution; LVOT—left ventricular outflow tract.

## Data Availability

Not applicable.
